# Adapting a Dementia Prevention Tool for Older Adults Living in Rural Communities: A Content Analysis

**DOI:** 10.1093/geroni/igaf122.021

**Published:** 2025-12-31

**Authors:** Jessica Cassidy, Kathy Lee

**Affiliations:** The University of Texas at Arlington, Arlington, Texas, United States; The University of Texas at Arlington, Arlington, Texas, United States

## Abstract

Older adults living in rural communities are at heightened risk of dementia; however, preventative strategies can reduce these risks. Before dementia prevention efforts can be effectively implemented in rural communities, gaps in the literature must be addressed by developing culturally relevant tools designed to measure the influence of community characteristics on local dementia-related health beliefs and behaviors. To achieve this objective, this study developed the Rural Older Adults Dementia prevention–Health Behaviors questionnaire (ROAD-HB) by applying a lifespan developmental approach to the Health Belief Model (HBM). The questionnaire consisted of three embedded instruments and was constructed following a stage development design. To explore participant perceptions of the ROAD-HB’s acceptability, usability, and content validity, cognitive interviews were conducted with 20 older adults from two rural Northeast Texas counties and themes were developed through a qualitative content analysis. There were six major themes identified which included: (1) Acceptability and positive feedback on ROAD-HB’s preventative focus; (2) Suggestions to improve inclusivity of questionnaire title; (3) Usability and Clarity Challenges due to low health literacy and a lack of familiarity with dementia; (4) Challenges with response options; (5) Differences between local rural culture health beliefs and those in items stems; and (6) Needs for Greater Specificity in Item Stems to Assess Health Behavior Norms. Overall, place-based factors were observed to shape participant’s dementia-related health beliefs and behaviors in multiple ways. The findings of this study may inform future dementia prevention efforts, which are urgently needed in rural communities.

